# Supplementary material for: Direct methane protonic ceramic fuel cells with self-assembled Ni-Rh bimetallic catalyst
Source: Nat Commun. 2023 Nov 18;14:7485. doi: 10.1038/s41467-023-43388-8 (PMC10657466; doi:10.1038/s41467-023-43388-8)
Supplement: Supplementary file 1 — Supplementary Information [file 41467_2023_43388_MOESM1_ESM.pdf]

Direct methane protonic ceramic fuel cells with self-assembled Ni-Rh bimetallic catalyst

Kyungpyo Hong[1$], Mingi Choi[2$], Yonggyun Bae[1,3], Jihong Min[1], Jaeyeob Lee[4], Donguk Kim[4], Sehee Bang[4], Han-Koo Lee[5]

Wonyoung Lee[4,6*], Jongsup Hong[1*]

$: These authors equally contributed to this work

Affiliations

[1] School of Mechanical Engineering, Yonsei University, Seoul, Republic of Korea

[2] Department of Future Energy Convergence, Seoul National University of Science & Technology, Seoul, Republic of Korea

[3] Department of Zero-carbon Fuel & Power Generation, Korea Institute of Machinery & Materials, Daejeon, Republic of Korea

[4] School of Mechanical Engineering, Sungkyunkwan University (SKKU), Suwon, Republic of Korea

[5] Pohang Accelerator Laboratory, Pohang University of Science and Technology (POSTECH), Pohang, Republic of Korea

[6] SKKU Institute of Energy Science and Technology (SIEST), Sungkyunkwan University, Suwon, Republic of Korea

---

[*] Corresponding author.
Email address: leewy@skku.edu (Wonyoung Lee)
[*] Corresponding author.
Email address: jongsup.hong@yonsei.ac.kr (Jongsup Hong)

Supplementary Figure 1. Single cell structure and synthesis process of the REF catalyst. (a) Schematic illustration of the fabrication of Ni-diffused BZCYYb backbone and subsequent Ni-exsolution during the reduction process. Cross-sectional SEM images for the structure of protonic ceramic fuel cell. The single cell before reduction (b), and after reduction (c). The magnified view of the cathode (PBSCF) (d), anode functional layer (Ni-BZCYYb) (e), and exsolved particles at the BZCYYb backbone (f).

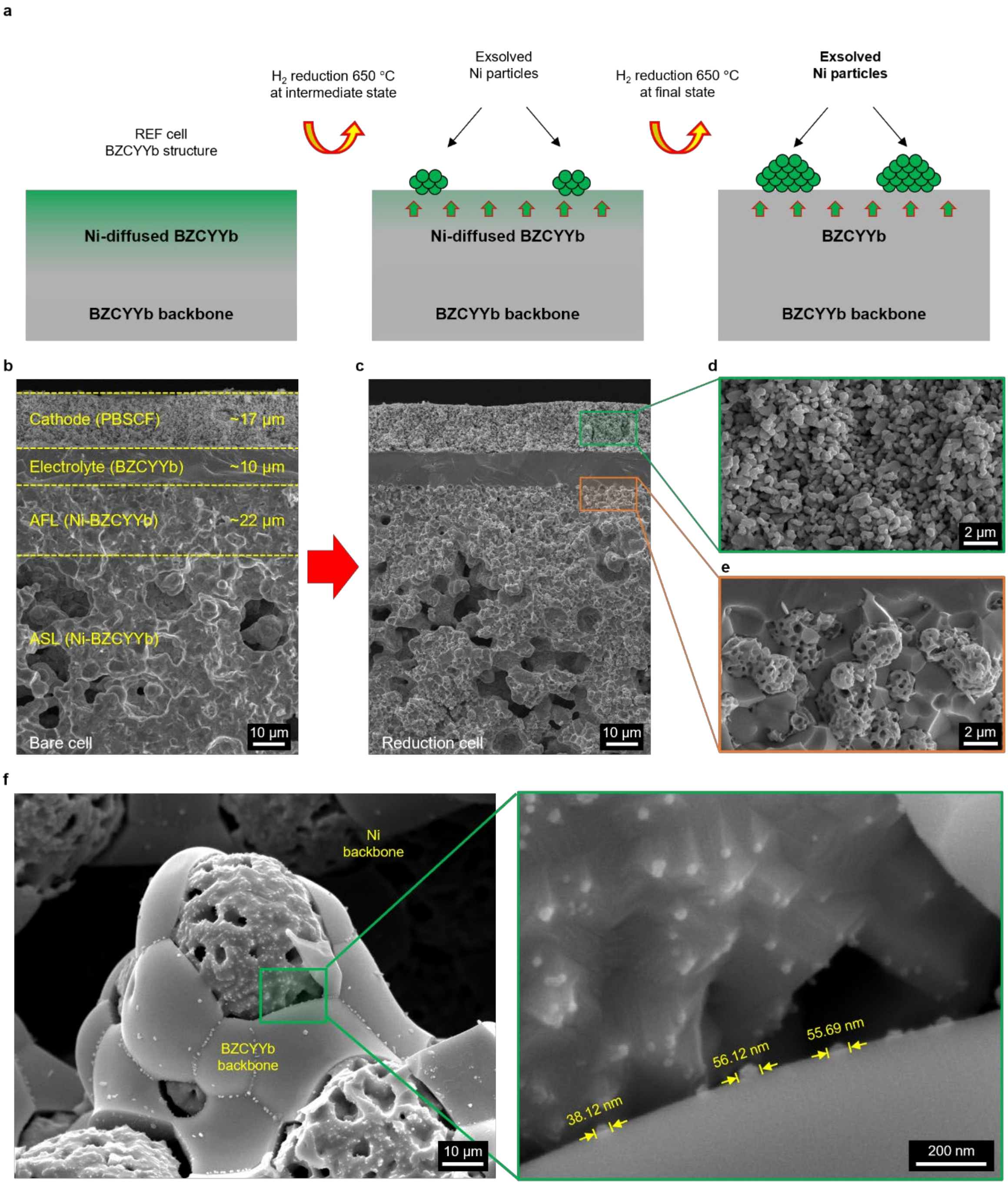

Supplementary Fig. 1(a) shows the schematic illustrations of the fabrication of a Ni-diffused BZCYYb backbone for a self-assembled Ni-Rh bimetallic catalyst. During the high-sintering process, Ni diffusion inside the perovskite oxide lattice occurs. Thus, Ni can be exsolved out of the lattice under H_2 reducing atmosphere. Using this mechanism, we fabricated the PCFC single cell in an anode-support configuration as shown in Supplementary Fig. 1(b-e). It shows the morphology of the fuel cell including specific microstructure. Onto the thick and porous support layer as a fuel electrode for gas reforming, which has a thickness of over 500 μm and pores of 5-13 μm, the anode functional layer, electrolyte, and cathode layer were fabricated with a thickness of ~22, ~10, and ~17 μm, respectively. It shows no-discernible structural deteriorations such as delamination between layers, micro/nano crack, and/or fracture, confirming the structural robustness of the fabricated cell after reduction under the H_2 environment. Through this mechanism, we could facilitate the Ni diffusion by controlling the sintering temperature toward the high temperature, resulting in a large number of exsolved Ni particles under a reducing atmosphere (See Supplementary Fig. 2). In the REF cell, the surface of the BZCYYb catalyst support is clean and smooth before reduction under the H_2 environment. However, after reduction, Ni nanoparticles (30–50 nm) are exsolved to the BZCYYb surface with a surface coverage of 11–13% as shown in Supplementary Fig. 1(f).

Supplementary Figure 2. Magnified images of cell structures by sintering temperature (1200-1500 ℃).

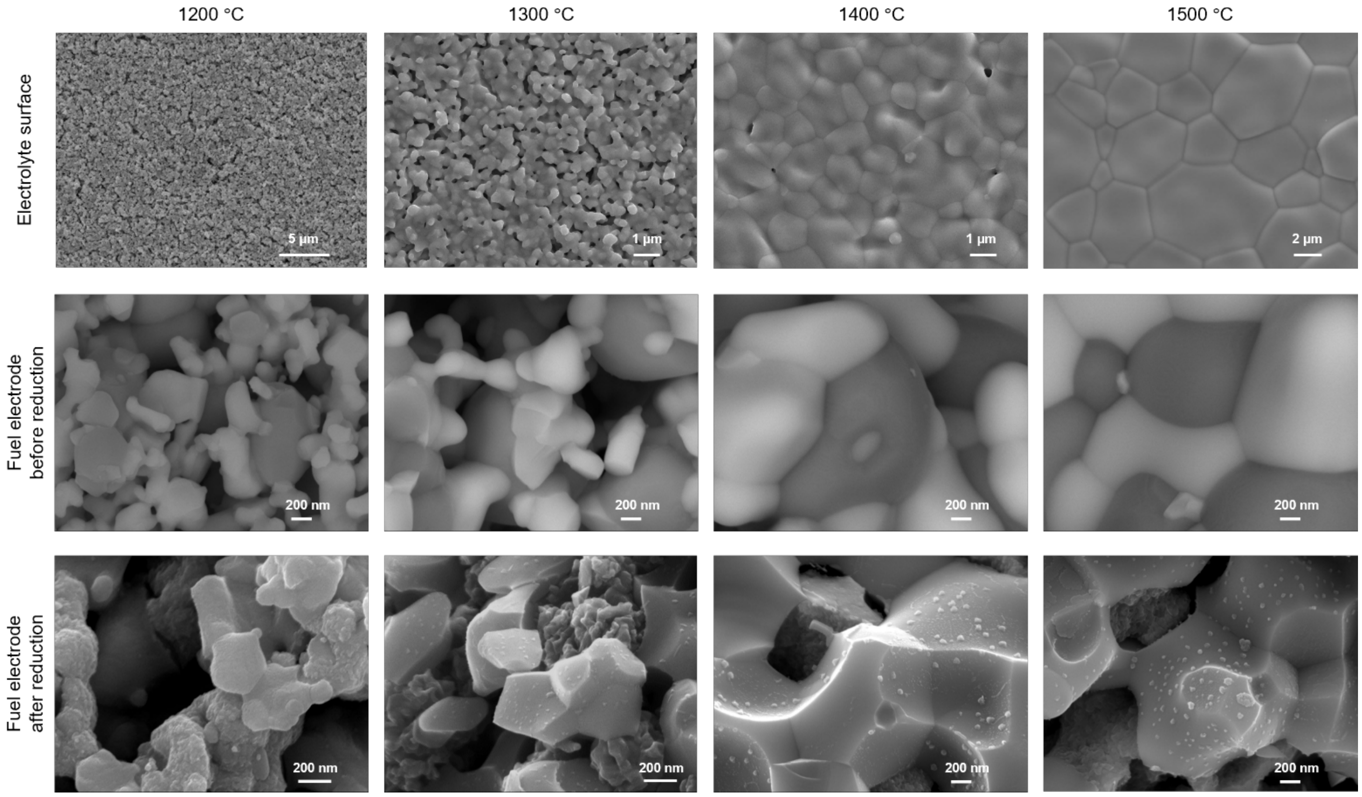

Supplementary Figure 3. Bimetallic catalyst formation of Ni-Rh cell. (a) XRD patterns and EDS mapping of Ni-Rh cell (b) before and (c) after reduction.

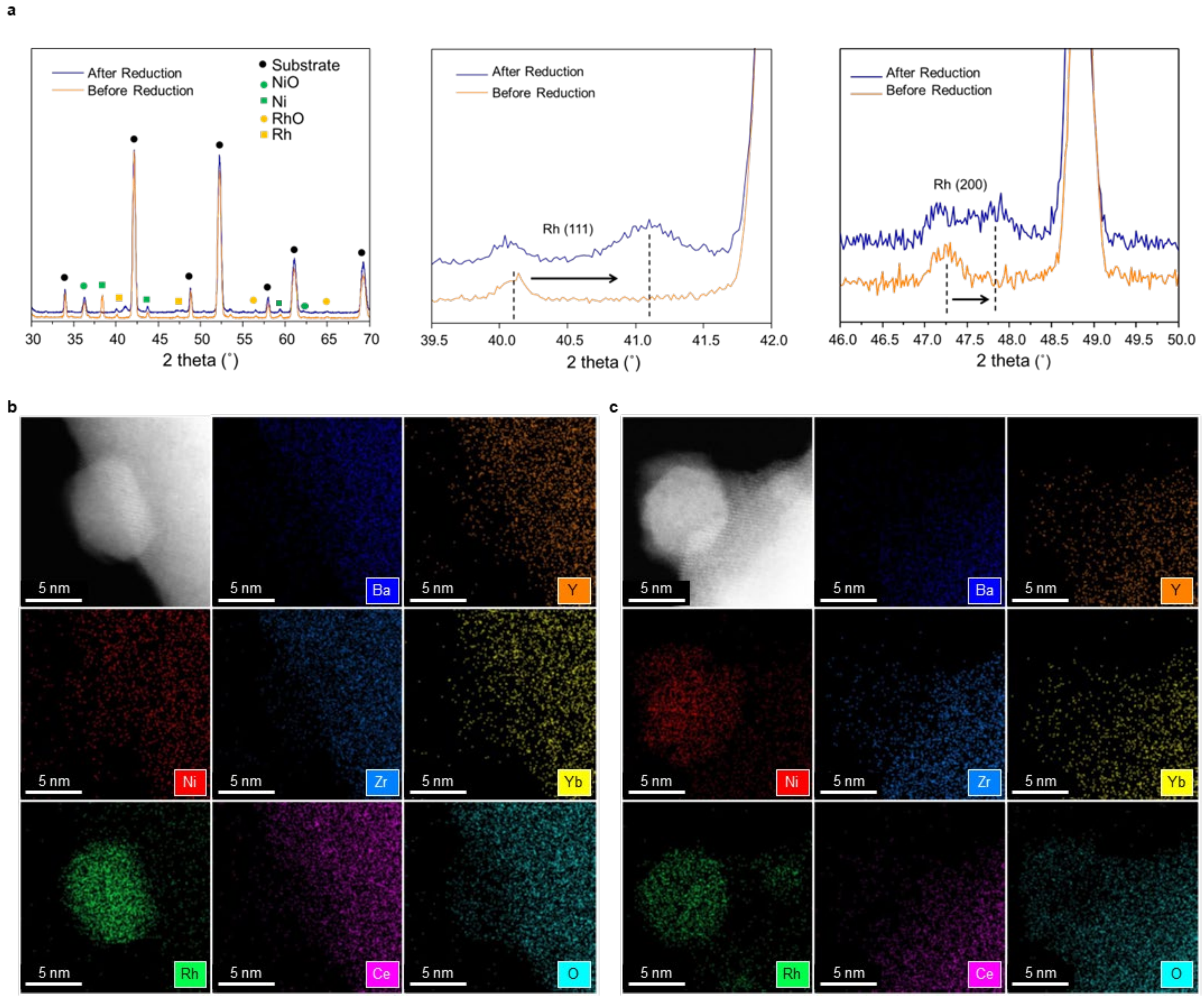

XRD patterns and EDS mapping in Supplementary Figure 3 show that Rh nanoparticles exist solely as a partially oxidized metallic phase (RhO and Rh) without mixing with Ni inside the BZCYYb lattice before reduction. Moreover, as shown in Supplementary Fig 3(a), Rh (111) and Rh (200) peak shifts toward the higher degree after reduction, confirming the change in the lattice parameters due to the alloy formation with Ni which has the smaller lattice constant.

Supplementary Figure 4. Electrochemical performance evaluation under the different fuel conditions including H_2 (a) and CH_4 (H_2O/CH_4) with S/C=2 (b) and S/C=1(c) in the temperature range of 650-450 °C for REF and Ni-Rh cells. The fuel conditions are 97% H_2 with 3% H_2O for H_2 (100 sccm), 25% CH_4, 50% H_2O and 25% Ar for S/C=2 (32 sccm), and 25% CH_4, 25% H_2O and 50% Ar for S/C=1 (32 sccm), respectively. Air is fed into the cathode as an oxidant (100 sccm).

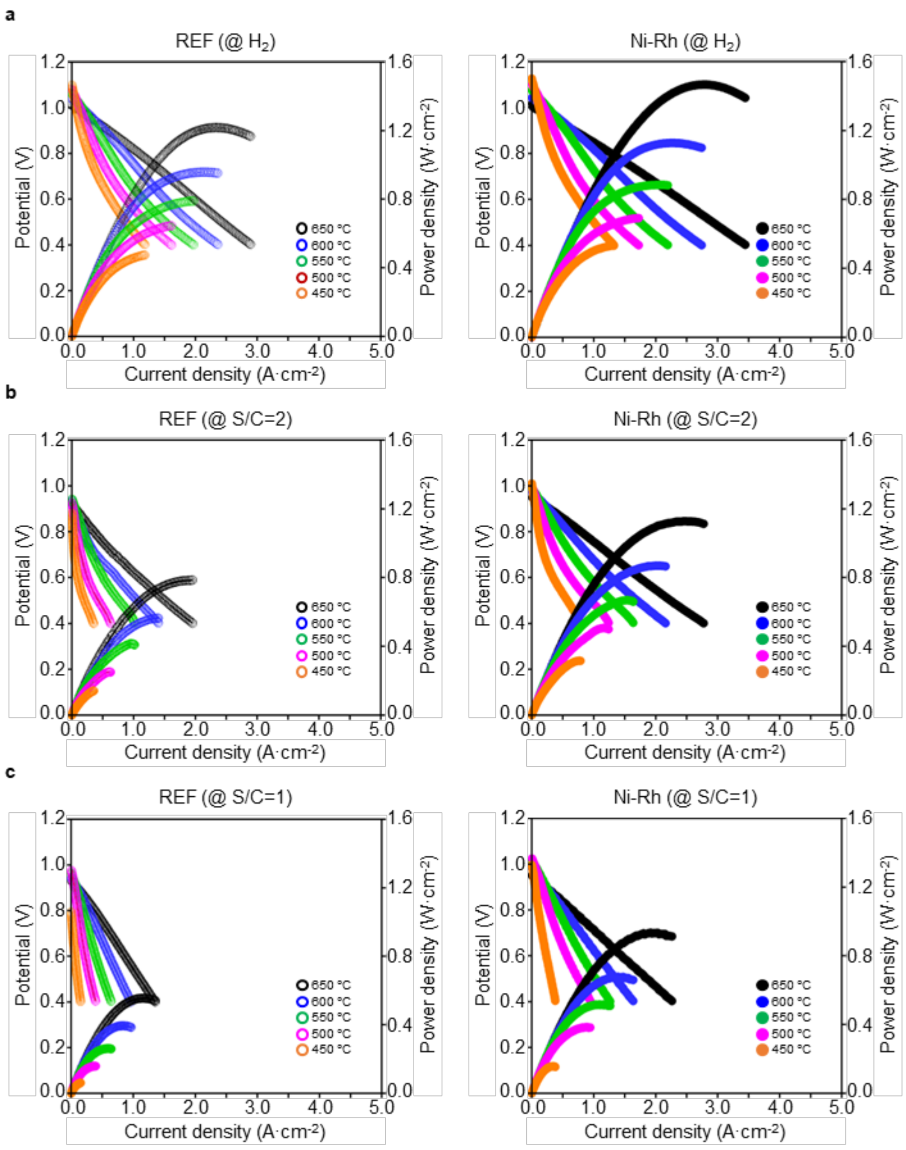

Supplementary Figure 5. Electrochemical impedance spectroscopy analysis under the different fuel conditions. (a) Nyquist plot, (b) Bode plot, and (c) DRT analysis for REF and Ni-Rh cells, respectively, under the different fuel conditions including H_2 and CH_4 (H_2O/CH_4) with S/C=2 and S/C=1 in the temperature range of 650-450 °C at OCV by the electrochemical impedance spectroscopy. The fuel conditions are 97% H_2 with 3% H_2O for H_2 (100 sccm), 25% CH_4, 50% H_2O and 25% Ar for S/C=2 (32 sccm), and 25% CH_4, 25% H_2O and 50% Ar for S/C=1 (32 sccm), respectively. Air is fed into the cathode as an oxidant (100 sccm).

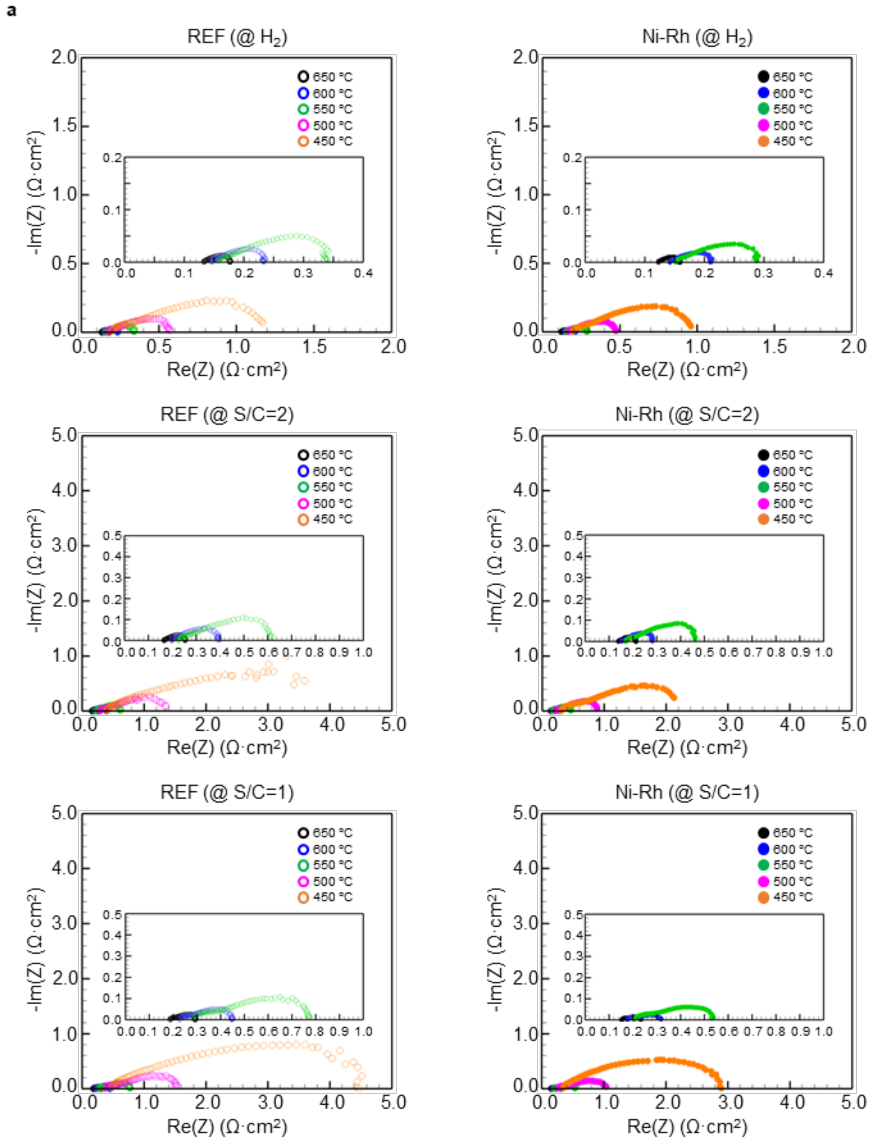

b

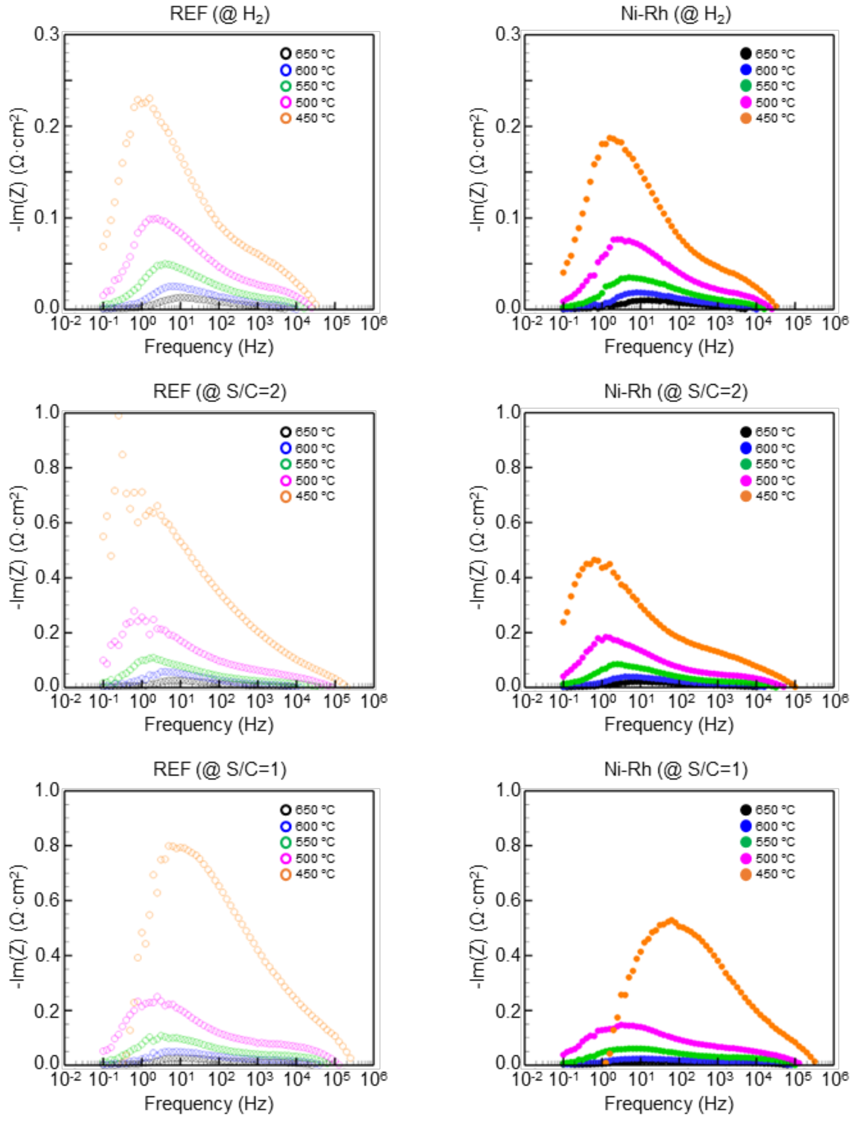
REF (@ H₂)
Ni-Rh (@ H₂)
REF (@ S/C=2)
Ni-Rh (@ S/C=2)
REF (@ S/C=1)
Ni-Rh (@ S/C=1)
650 °C
600 °C
550 °C
500 °C
450 °C
-Im(Z) (Ω·cm²)
Frequency (Hz)

c

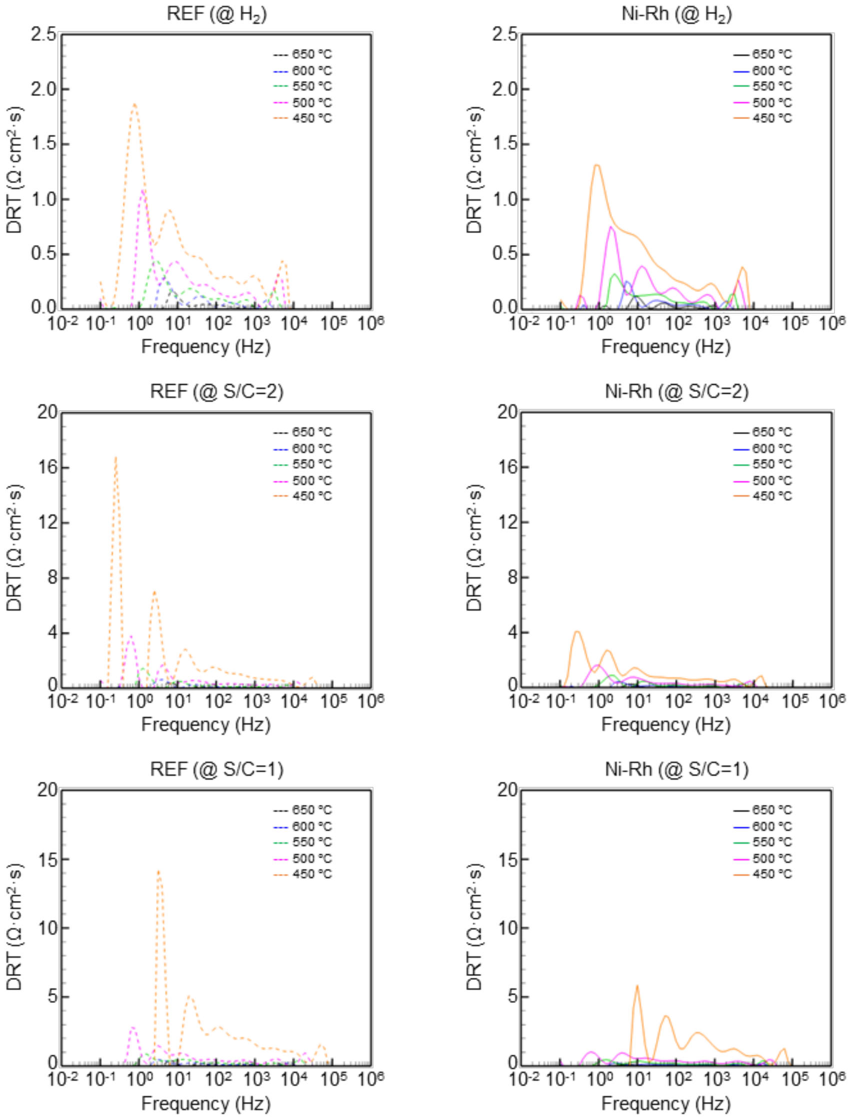

REF (@ H_2)
Ni-Rh (@ H_2)
REF (@ S/C=2)
Ni-Rh (@ S/C=2)
REF (@ S/C=1)
Ni-Rh (@ S/C=1)
650 °C
600 °C
550 °C
500 °C
450 °C
DRT (Ω·cm²·s)
Frequency (Hz)

Supplementary Table 1. OCVs and MPDs under the CH_4-fueled operation (S/C=2, S/C=1) in the temperature range of 650-450 °C for REF cell (a) and Ni-Rh cell (b).

**Steam to carbon ratio**	**Temperature (°C)**	**MPD (W·cm^{-2})** REF / **Ni-Rh**	**Measured OCV (V)** REF / **Ni-Rh**
S/C = 2	650	0.78 / **1.13**	0.917 / **0.949**
	600	0.56 / **0.87**	0.938 / **0.973**
	550	0.42 / **0.67**	0.942 / **0.991**
	500	0.25 / **0.50**	0.925 / **1.005**
	450	0.14 / **0.32**	0.880 / **1.010**
S/C = 1	650	0.56 / **0.93**	0.932 / **0.951**
	600	0.39 / **0.38**	0.944 / **0.983**
	550	0.26 / **0.52**	0.963 / **1.017**
	500	0.16 / **0.39**	0.976 / **1.024**
	450	0.06 / **0.16**	0.794 / **0.996**

Supplementary Table 1 exhibits the maximum power density (MPD) and measured OCV under the CH_4-fueled operation. There is a difference in performance and OCV between the REF cell and the Ni-Rh cell, which is related to the hydrogen partial pressure. Performance increases with a higher partial pressure of H_2[1]. In addition, according to the Nernst equation ($E_T = E_T^{\circ} + \left(\frac{RT}{2F}\right)\ln\left(P_{H2} \cdot P_{O2}^{1/2}/P_{H2O}\right)$), the OCV increases with higher partial pressure of H_2 and lower partial pressure of H_2O. Through these trends, it can be predicted that the CH_4 and H_2O activations (methane steam reforming) of the Ni-Rh cell occur more than those of the REF cell.

Supplementary Figure 6. Electrochemical performance evaluation under different partial pressures of H_2 with 3% wet condition in the temperature range of 650-450 °C for REF cell (a) and Ni-Rh cell (b). The fuel conditions are 97% H_2 with 3% H_2O for 100% H_2, 77.6% H_2, 19.4% Ar with 3% H_2O for 80% H_2, 58.2% H_2, 38.8% Ar with 3% H_2O for 60% H_2, 38.8% H_2, 58.2% Ar with 3% H_2O for 40% H_2, 19.4% H_2, 77.6% Ar with 3% H_2O for 20% H_2, respectively, under same flow rate of 100 sccm. Air is fed into the cathode as an oxidant (100 sccm).

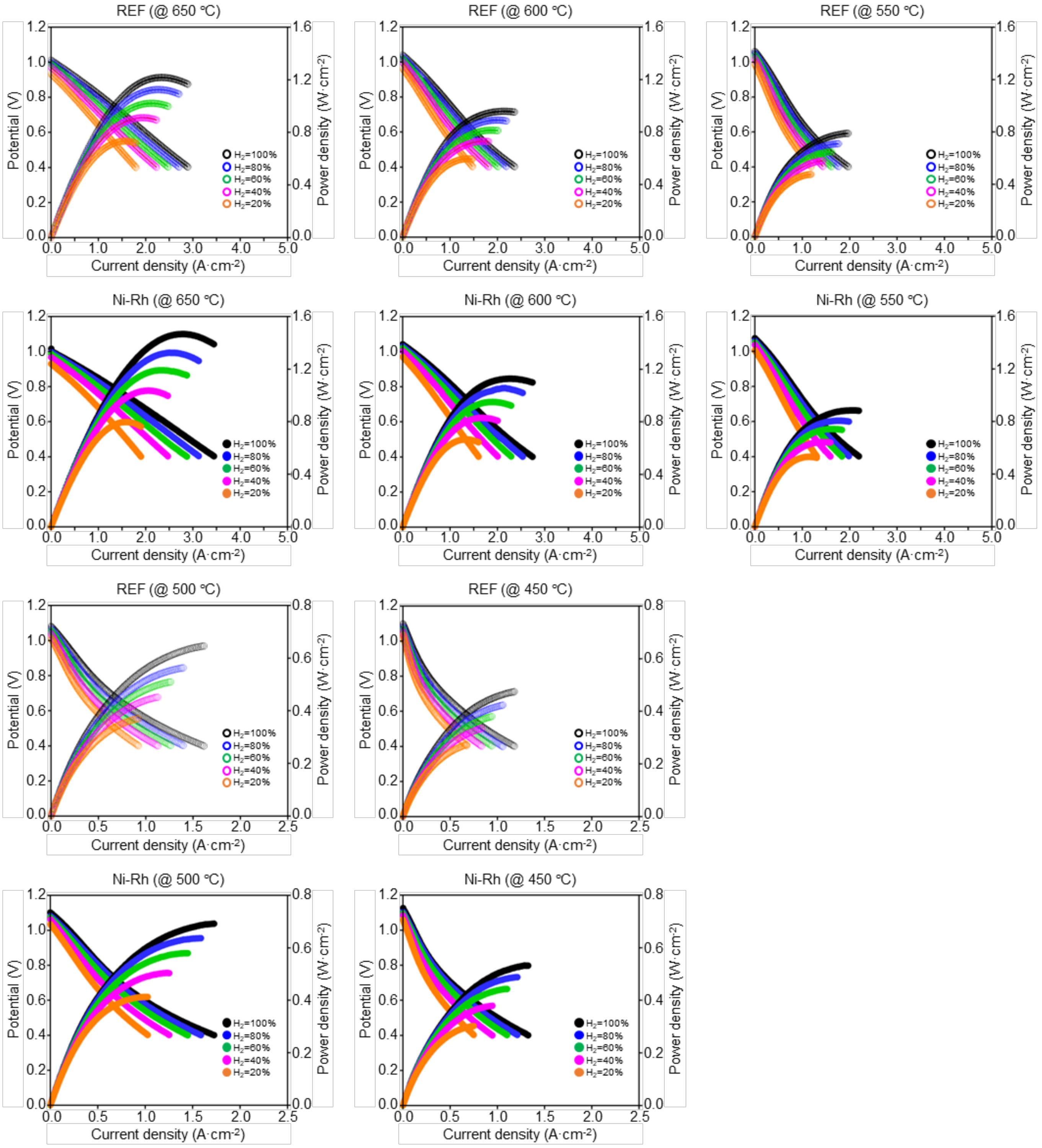

Supplementary Figure 7. Nyquist plot for REF and Ni-Rh cells under different partial pressure of H_2 with 3% wet condition in the temperature range of 650-450 °C at OCV. The fuel conditions are 97% H_2 with 3% H_2O for 100% H_2, 77.6% H_2, 19.4% Ar with 3% H_2O for 80% H_2, 58.2% H_2, 38.8% Ar with 3% H_2O for 60% H_2, 38.8% H_2, 58.2% Ar with 3% H_2O for 40% H_2, 19.4% H_2, 77.6% Ar with 3% H_2O for 20% H_2, respectively, under same flow rate of 100 sccm. Air is fed into the cathode as an oxidant (100 sccm).

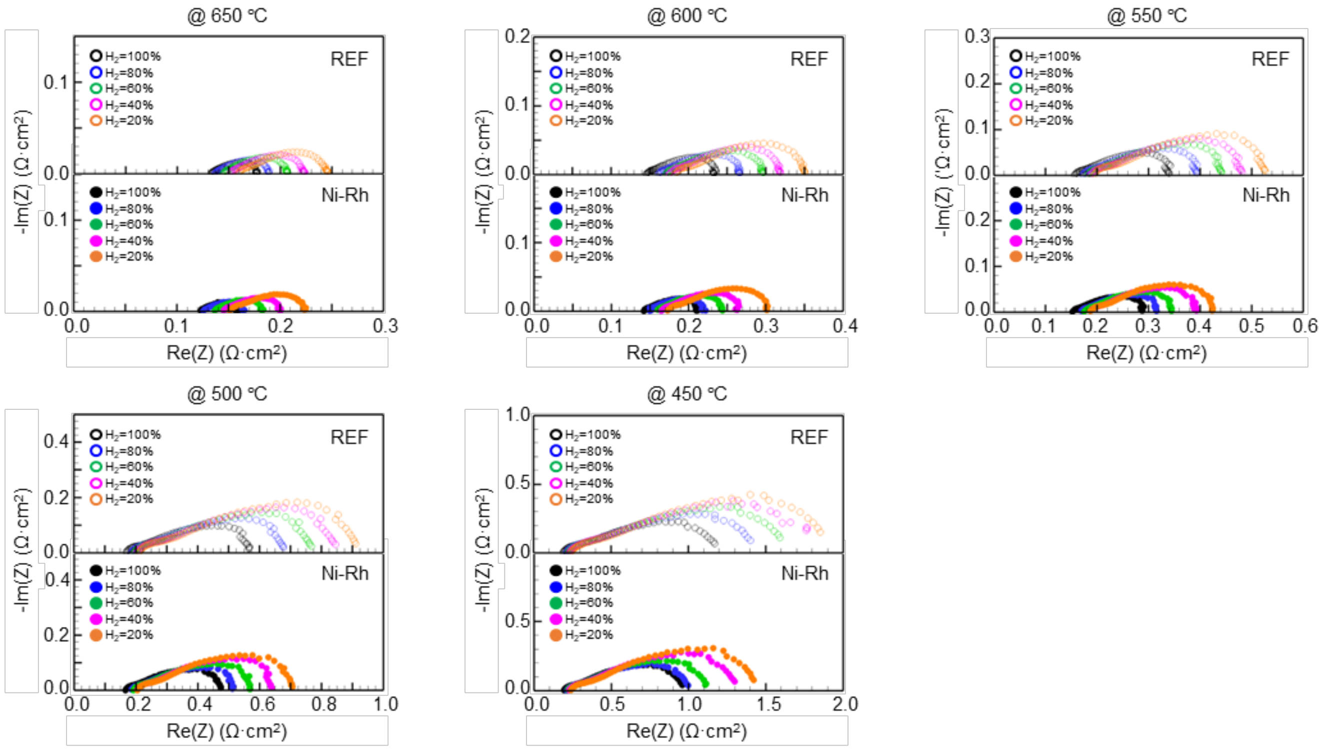

Supplementary Fig. 7 shows the ohmic and polarization resistance for REF and Ni-Rh cells under different partial pressures of H_2 by Nyquist plot. In this figure, the ohmic and polarization resistance decrease as the partial pressure of H_2 is lowered because the amount of H_2 that can react electrochemically per unit effective reaction area is reduced.

Supplementary Table 2. OCVs and MPDs under different partial pressures of H_2 with 3% wet condition in the temperature range of 650-450 °C for REF cell (a) and Ni-Rh cell (b).

**Temperature (°C)**	**Partial pressure of H_2 (%) with 3% wet**	**MPD ($W \cdot cm^{-2}$)** REF / **Ni-Rh**	**Measured OCV (V)** REF / **Ni-Rh**	**Theoretical OCV (V)**
650	100	1.22 / **1.47**	1.011 / **1.015**	1.128
	80	1.12 / **1.32**	0.999 / **0.996**	1.119
	60	1.02 / **1.19**	0.987 / **0.985**	1.107
	40	0.91 / **1.04**	0.969 / **0.967**	1.091
	20	0.73 / **0.80**	0.932 / **0.929**	1.064
600	100	0.96 / **1.13**	1.039 / **1.041**	1.135
	80	0.89 / **1.06**	1.028 / **1.030**	1.127
	60	0.81 / **0.95**	1.015 / **1.019**	1.116
	40	0.73 / **0.83**	0.997 / **1.003**	1.101
	20	0.60 / **0.67**	0.963 / **0.970**	1.075
550	100	0.79 / **0.88**	1.061 / **1.074**	1.144
	80	0.71 / **0.81**	1.050 / **1.059**	1.136
	60	0.65 / **0.74**	1.037 / **1.049**	1.126
	40	0.58 / **0.64**	1.019 / **1.037**	1.111
	20	0.48 / **0.53**	0.989 / **1.006**	1.087
500	100	0.65 / **0.69**	1.080 / **1.101**	1.152
	80	0.56 / **0.64**	1.067 / **1.081**	1.145
	60	0.51 / **0.58**	1.055 / **1.072**	1.135
	40	0.45 / **0.50**	1.037 / **1.062**	1.122
	20	0.37 / **0.41**	1.013 / **1.034**	1.098
450	100	0.47 / **0.53**	1.098 / **1.125**	1.161
	80	0.42 / **0.49**	1.080 / **1.103**	1.154
	60	0.38 / **0.44**	1.068 / **1.094**	1.145
	40	0.33 / **0.38**	1.054 / **1.084**	1.132
	20	0.27 / **0.30**	1.036 / **1.059**	1.110

Supplementary Note 1.

Electrochemical performance evaluation under the CH_4-fueled with S/C=1 in the temperature range of 650-450 °C for REF and Ni-Rh cells

Under CH_4 fuel condition (S/C=1), the Ni-Rh cell showed ~1.68-fold higher MPDs at 650 °C (~0.56 $W{\cdot}cm^{-2}$ for REF cell and ~0.93 $W{\cdot}cm^{-2}$ for Ni-Rh cell, respectively), and ~2.42-fold higher MPDs at 500 °C (~0.16 $W{\cdot}cm^{-2}$ for REF cell and ~0.39 $W{\cdot}cm^{-2}$ for Ni-Rh cell, respectively). The relative performance ratio between MPDs under CH_4 and H_2 ($MPD_{CH4(S/C=1)}/MPD_{H2}$) were ~0.45 and ~0.64 at 650 °C, and ~0.24 and ~0.56 at 500 °C for REF and Ni-Rh cells, respectively.

Supplementary Table 3. Comparison of the maximum power density (MPD) and area-specific polarization resistance ($ASR_{electrode}$) with the reported PCFCs and SOFCs.

Cell components		Fuel composition (%)	Temperature (°C)	MPD ($W \cdot cm^{-2}$)	$ASR_{electrode}$ (Ωcm^2)	Note	[Ref]
Cathode	$PrBa_{0.5}Sr_{0.5}Co_{1.5}Fe_{0.5}O_{5+\delta}$		650	1.13	0.07	1) Anode: self-assembled Ni-Rh bimetallic fuel electrode by extremely small amount one-step infiltration	This study
			600	0.87	0.13		
Electrolyte	$BaZr_{0.4}Ce_{0.4}Y_{0.1}Yb_{0.1}$	CH_4(25) / H_2O(50) / Ar	550	0.67	0.29		
			500	0.50	0.67		
Anode (AFL/ASL)	$Ni\text{-}BaZr_{0.4}Ce_{0.4}Y_{0.1}Yb_{0.1}O_{3-\delta}$		450	0.32	1.89		
Cathode	$PrBa_{0.5}Sr_{0.5}Co_{1.5}Fe_{0.5}O_{5+\delta}$		650	0.93	0.08		
			600	0.68	0.14		
Electrolyte	$BaZr_{0.4}Ce_{0.4}Y_{0.1}Yb_{0.1}$	CH_4(25) / H_2O(25) / Ar	550	0.52	0.33		
			500	0.39	0.78		
Anode	$Ni\text{-}BaZr_{0.4}Ce_{0.4}Y_{0.1}Yb_{0.1}O_{3-\delta}$		450	0.16	2.26		
Cathode	$PrBa_{0.5}Sr_{0.5}Co_{1.5}Fe_{0.5}O_{5+\delta}$		650	0.78	0.09	Reference cell	
			600	0.56	0.20		
Electrolyte	$BaZr_{0.4}Ce_{0.4}Y_{0.1}Yb_{0.1}$	CH_4(25) / H_2O(50) / Ar	550	0.42	0.40		
			500	0.25	1.11		
Anode	$Ni\text{-}BaZr_{0.4}Ce_{0.4}Y_{0.1}Yb_{0.1}O_{3-\delta}$		450	0.14	3.32		
Cathode	$PrBa_{0.5}Sr_{0.5}Co_{1.5}Fe_{0.5}O_{5+\delta}$		650	0.56	0.11		
			600	0.39	0.22		
Electrolyte	$BaZr_{0.4}Ce_{0.4}Y_{0.1}Yb_{0.1}$	CH_4(25) / H_2O(25) / Ar	550	0.26	0.49		
			500	0.16	1.23		
Anode	$Ni\text{-}BaZr_{0.4}Ce_{0.4}Y_{0.1}Yb_{0.1}O_{3-\delta}$		450	0.06	4.25		
Cathode	$BaCo_{0.4}Fe_{0.4}Zr_{0.1}Y_{0.1}O_{3-\delta}$		650	-	-		
			600	0.36	0.28		
Electrolyte	$BaZr_{0.8}Y_{0.2}O_{3-\delta}$	CH_4(33.3) / H_2O(66.7)	550	0.31	-	1) Anode: exsolution of Ni NPs on the BZY20 surface	2
			500	0.23	-		
Anode	$Ni\text{-}BaZr_{0.8}Y_{0.2}O_{3-\delta}$ (+1wt% NiO)		450	-	-		
Cathode	$BaCo_{0.4}Fe_{0.4}Zr_{0.1}Y_{0.1}O_{3-\delta}$		650	-	-		
			600	0.29	-		
Electrolyte	$BaZr_{0.8}Y_{0.2}O_{3-\delta}$ (1wt% NiO)	CH_4(28.6) / H_2O(71.4)	550	0.22	-	1) Electrolyte: lower electronic leak by sintering 1wt% NiO with electrolyte	3
			500	0.14	-		
Anode	$Ni\text{-}BaZr_{0.8}Y_{0.2}O_{3-\delta}$		450	-	-		
Cathode	$PrNi_{0.7}Co_{0.3}O_{3-\delta}$		650	-	-		
			600	0.45	-		
Electrolyte	$BaZr_{0.1}Ce_{0.7}Y_{0.1}Yb_{0.1}O_{3-\delta}$	CH_4 (with 3% H_2O)	550	-	-	1) Cathode: material optimization (PrNixCo1-xO3-δ), 3-D microstructure<br>2) Electrolyte: improvement adhesion between oxygen electrode and electrolyte by acid treatment	4
			500	-	-		
Anode	$Ni\text{-}BaZr_{0.1}Ce_{0.7}Y_{0.1}Yb_{0.1}O_{3-\delta}$		450	-	-		
Cathode	$PrBa_{0.5}Sr_{0.5}Co_{1.5}Fe_{0.5}O_{5+\delta}$		650	-	-		
			600	-	-		
Electrolyte	$Ce_{0.8}Sm_{0.2}O_2$	CH_4 (with 3.5% H_2O)	550	-	-	1) Cathode: PBSCF nanofibers coated with NPs of PrOx<br>2) Anode: anode reforming layer ($Ce_{0.90}Ni_{0.05}Ru_{0.05}O_2$)	5
			500	0.37	-		
Anode	$Ni\text{-}BaZr_{0.1}Ce_{0.7}Y_{0.1}Yb_{0.1}O_{3-\delta}$		450	-	-		
Cathode	$BaCe_{0.55}Zr_{0.3}Y_{0.1}5_{O3-\delta}$ - $Ba_{0.95}La_{0.05}Fe_{0.8}Zn_{0.2}O_{3-\delta}$		650	-	-		
			600	0.61	-		
Electrolyte	$BaZr_{0.5}Ce_{0.3}Y_{0.1}Yb_{0.1}O_{3-\delta}$	CH_4 (with 20% H_2O)	550	-	-	1) Cathode: $BaCo_{0.4}Fe_{0.4}Zr_{0.1}Y_{0.1}O_{3-\delta}$ infiltration into the BCZY-BLFZ	6
			500	-	-		
Anode	$Ni\text{-}BaZr_{0.5}Ce_{0.3}Y_{0.1}Yb_{0.1}O_{3-\delta}$		450	-	-		
Cathode	$BaZr_{0.1}Ce_{0.7}Y_{0.1}O_{3-\delta}$ - $LaSr_3Co_{1.5}Fe_{1.5}O_{10-\delta}$		650	0.98	0.19		
			600	0.65	0.33		
Electrolyte	$BaZr_{0.1}Ce_{0.7}Y_{0.1}Yb_{0.1}O_{3-\delta}$	CH_4 (with 3% H_2O)	550	0.51	0.36	1) Electrolyte: thin film (3 μm) by e-beam deposition technique<br>2) Anode: anode reforming layer ($LaNi_{0.6}Co_{0.4}O_3$)	7
			500	0.40	-		
Anode	Ni- $BaZr_{0.1}Ce_{0.7}Y_{0.1}Yb_{0.1}O_{3-\delta}$		450	-	-		
Cathode	$(La_{0.60}Sr_{0.40})_{0.95}Co_{0.20}Fe_{0.80}O_{3-\delta}$		650	0.13	0.67		
			600	0.10	1.32		
Electrolyte	$BaZr_{0.8}Y_{0.2}O_{3-\delta}$ (+1wt% NiO)	CH_4(33) / H_2O(33) / N_2	550	0.06	4.59	1) Cathode: $PrNi_{0.5}Mn_{0.5}O_3\text{-}PrO_x$ (PNM) impregnation into the LSCF by vacuum machine	8
			500	-	-		
Anode	Ni- $BaZr_{0.8}Y_{0.2}O_{3-\delta}$		450	-	-		
Cathode	$La_{0.6}Sr_{0.4}Co_{0.2}Fe_{0.8}O_{3-\delta}$ - Gd doped CeO_2 (GDC)		650	-	-		
			600	-	-		
Electrolyte	Gd doped CeO_2 (GDC)	CH_4(10) / H_2O(60) / N_2	554	0.45	0.45	1) Anode: anode functional layer (pure-ceria CeO_2) for reforming reaction	9
			503	0.30	1.38		
Anode tube	Ni-Gd doped CeO_2 (GDC)		449	0.11	-		
Cathode	$Sm_{0.5}Sr_{0.5}CoO_3$ - Gd doped CeO_2 ($Ce_{0.9}Gd_{0.1}O_{1.95}$)		650	0.57	0.10		
			600	0.52	0.14		
Electrolyte	Gd doped CeO_2 ($Ce_{0.9}Gd_{0.1}O_{1.95}$)	CH_4 (with 3% H_2O)	550	0.34	0.47	1) Electrolyte: thin film (20 μm) by applying copressing<br>2) Anode: solution impregnation process	10
			500	0.17	1.92		
Anode	Ni-Gd doped CeO_2 ($Ce_{0.9}Gd_{0.1}O_{1.95}$)		450	-	-		

Supplementary Figure 8. Nyquist plots of symmetric cell of REF and Ni-Rh fuel electrode under (a) H_2 and (b) CH_4/H_2O (S/C=1) environments at 500 °C. Area-specific polarization resistances according to different frequency ranges, high (> 10^3 Hz), medium (10–10^3 Hz), and low (< 10 Hz) frequencies under different fuel conditions (c) H_2, and (d) CH_4(S/C=1)).

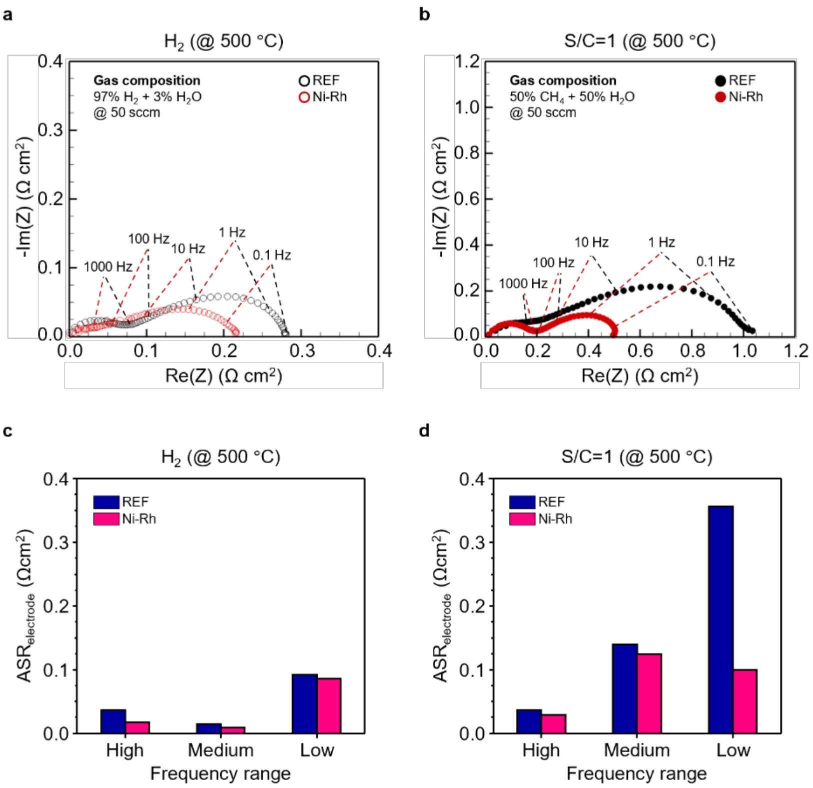

We conducted additional symmetric cell analysis of the REF and Ni-Rh fuel electrode under H_2 and CH_4/H_2O environments to further verify the effect of Ni-Rh on the decrease in ASR_p, as shown in Supplementary Fig. 8. Under the H_2 environment, the Ni-Rh cell shows the slight decrease in ASR_p (0.108 ohm·cm^{-2} at 500 ˚C) compared to that of the REF cell (0.140 ohm·cm^{-2} at 500 ˚C). Under the CH_4/H_2O environment, ASR_p of the Ni-Rh cell shows a ~2-fold smaller value (0.249 ohm·cm^{-2} at 500 ˚C) than that of the REF cell (0.565 ohm·cm^{-2} at 500 ˚C), which is more significant difference compared to that under H_2. Note that we divided the values of ASR_p in Supplementary Fig. 8 by two since they are the symmetric cells. Especially, frequency analysis in Supplementary Fig. 8(c-d) show the same trend with that of the single cell in Figure 2(g). The Ni-Rh cell exhibits slightly lower resistances than the REF cell at high frequencies under all fuel conditions due to the enhanced electrochemical activity for charge transfer at the TPB and the overall hydrogen oxidation reactions (HOR) at the fuel electrode. Under CH_4 fuel condition, the medium frequency resistances significantly increase by a similar magnitude in both REF and Ni-Rh cells due to the slow gas–solid interaction caused by the reduced partial pressure of H_2 and the sluggish CH_4 adsorption. However, although the low-frequency resistances for the REF cell significantly increase by sluggish gas reforming under CH_4 operation, those for the Ni-Rh cell almost remain unchanged. Therefore, it confirms that Ni-Rh slightly improves the

electrochemical reaction for HOR under the H_2 environment and improves the gas reforming and gas-solid interaction under the CH_4/H_2O environment. Higher CH_4 conversion of the Ni-Rh fuel electrode at OCV condition measured by GC (Supplementary Fig. 9) further supports that Ni-Rh fuel electrode improves the sluggish gas–solid interaction and gas reforming.

Supplementary Note 2.

Catalytic activity and thermochemical analysis

The product gases exiting the anode outlet at the cell station were measured by gas chromatography (GC, Agilent 7890B) equipped with a dual TCD at OCV condition to analyze the catalytic activity but not to be affected by the electrochemical reaction. The dual TCD was connected to two columns (HP-PLOT 5A: H_2, CO, and HP-PLOT-Q: CH_4, CO_2) that separated the mixed gases.

To confirm the correlation between catalytic activity and performance, the methane conversion (CH_4 conv.) and hydrogen production rate (H_2 prod.) were calculated through the dry gas composition from GC. The methane conversion and hydrogen production rate were expressed using Eq. 1 and Eq. 2, respectively. The activation energy by the Arrhenius equation was analyzed using Eq. 3 to confirm the effect of methane conversion and hydrogen production rate as the catalyst was applied.

$$CH_{4,conv.} = \frac{F_{CH_4} - Q_{CH_4}}{F_{CH_4}} \times 100\ [\%] \qquad \text{(Eq. 1)}$$

$$H_{2,prod.} = Q_{H_2}\ [\mathrm{sccm}] = Q_{H_2} \times 10^{-3} \left[\frac{\mathrm{L}}{\mathrm{min}}\right] = Q_{H_2} \times \frac{10^{-3}}{22.4} \left[\frac{\mathrm{mol}}{\mathrm{min}}\right] = Q_{H_2} \times \frac{10^{-3}}{22.4\times 60} \left[\frac{\mathrm{mol}}{\mathrm{s}}\right] \qquad \text{(Eq. 2)}$$

$$\ln\left(CH_{4,conv.}\ or\ H_{2,prod.}\right) = \left(-\frac{E_a}{RT}\right) + \mathrm{Const} \qquad \text{(Eq. 3)}$$

where $CH_{4,conv.}$: methane conversion [%]; $H_{2,prod.}$: hydrogen production rate [mol/s]; $\ln\left(CH_{4,conv.}\ or\ H_{2,prod.}\right)$: Arrhenius equation; F_i: the volumetric flow rate of gas species i at the inlet [sccm]; Q_i: the volumetric flow rate of gas species i at the outlet [sccm]; E_a: activation energy [kJ/mol]; R: universal gas constant [J/mol/K]; and T: temperature [K]

The CH_4 conversion and H_2 production rate under S/C=2 and S/C=1 condition were evaluated by GC measurement during cell operation to quantitatively compare the catalytic activity of REF and Ni-Rh cell, as shown in Supplementary Fig. 9. In the case of S/C=2 condition, the CH_4 conversion of Ni-Rh cell (~87.2% at 650 °C and ~46.3% at 500 °C) are not only higher than that of REF cell (~73.7% at 650 °C and ~30.6% at 500 °C) but also close to equilibrium (calculated by Cantera in Supplementary Fig. 10). In addition, the results of H_2 production rate also follow the trend of those of CH_4 conversion (Ni-Rh cell: ~51.0×10^{-6} mol/s at 650 °C and ~34.1×10^{-6} mol/s at 500 °C; REF cell: ~43.2×10^{-6} mol/s at 650 °C and ~20.4×10^{-6} mol/s at 500 °C, respectively). The reason for the higher activity of the Ni-Rh cell than that of the REF cell is that the formation of Ni-Rh bimetallic catalyst produces a high active surface area with nano-sized particles (5~10 nm) as well as low activation energys (E_a). The activation energies of Ni-Rh cell (CH_4 conv. ~26.6 kJ/mol and H_2 prod. ~20.7 kJ/mol) are lower than those of REF

(CH_4 conv. ~34.8 kJ/mol and H_2 prod. ~29.4 kJ/mol), indicating that the Ni-Rh bimetallic alloy catalyst improves the activation energy for CH_4 reforming and H_2 production at the fuel electrode[11]. In the case of S/C = 1 condition, the trend of catalytic activity was also consistent (CH_4 conv., H_2 prod., E_a, constant value), although lower than that of S/C = 2 condition due to the thermodynamic limit of methane steam reforming. When all results of CH_4 operating conditions are considered, we could confirm that activated CH_4 reforming at Ni-Rh cell electrode provides higher P_{H2} than that at REF cell (Supplementary Fig. 11(a)). Moreover, relative catalytic activity improvement of Ni-Rh cell compared to that of REF cell for CH_4 conversion and H_2 production is more pronounced under higher P_{H2O} condition; ([CH_4 conv.$_{Ni-Rh}$]/[CH_4 conv.$_{REF}$]) = ~1.62 for S/C=2, and ~1.53 for S/C=1, ([H_2 prod.$_{Ni-Rh}$]/[H_2 prod.$_{REF}$]) = ~1.67 for S/C=2, and ~1.48 for S/C=1 at 500 °C. It might be due to the high reaction kinetics of Rh for H_2O dissociation[12], which significantly drives the steam methane reforming. Therefore, the formation of Ni-Rh bimetallic catalyst can improve the catalytic activity of Ni by alloying Ni with Rh regardless of any operating conditions[11].

In order to confirm the correlation between these catalytic activities and fuel cell performance, the gas composition including P_{H2O} present inside the cell was derived by considering the C-H-O balance based on the GC data (Supplementary Fig. 11(b)). As shown in Supplementary Fig. 12(a, b), the maximum power densities of REF and Ni-Rh cells are plotted for different partial pressure of H_2 and temperatures. Based on these results, it can be seen that the H_2 partial pressure is very important in the electrochemical performance when the results of the H_2 partial pressure (x-axis) inside the cell and MPD (y-axis) obtained from the CH_4 fuel operation are applied. The Ni-Rh cell in Supplementary Fig. 12(d, f) follow the calculated lines obtained from the results of partial pressure of H_2. However, the REF cell in Supplementary Fig. 12(c, e) does not catch up overall under the S/C=2 condition and even get further away under the S/C=1 condition. Because the Ni-Rh cell can sufficiently provide the H_2 without kinetic limitations, and there is little difference of low frequency resistance (Fig. 2(g)) between H_2 fuel and CH_4 fuels (S/C=2, S/C=1) operation, which results in similar MPDs (P_{H2}, S/C=2, S/C=1). On the other hand, the REF cell demonstrates significantly lower MPDs compared to that of the P_{H2} condition because the sluggish reaction kinetics of Ni substantially results in a high difference of low-frequency resistance between H_2 fuel and CH_4 fuels. Therefore, we can conclude that the Ni-Rh bimetallic catalyst has high catalytic activity with low activation energies, which significantly improves CH_4 activation and H_2 supply.

Supplementary Figure 9. Catalytic activity of REF and Ni-Rh cells for methane steam reforming in the temperature range of 650-450 °C at OCV. CH_4 conversion and H_2 production rate of REF and Ni-Rh cells and their Arrhenius behaviors under (a-c) S/C=2 and (d-f) S/C=1. The fuel conditions for fuel electrode are 25% CH_4, 50% H_2O and 25% Ar for S/C=2, and 25% CH_4, 25% H_2O and 50% Ar for S/C=1, respectively, under same flow rate of 32 sccm.

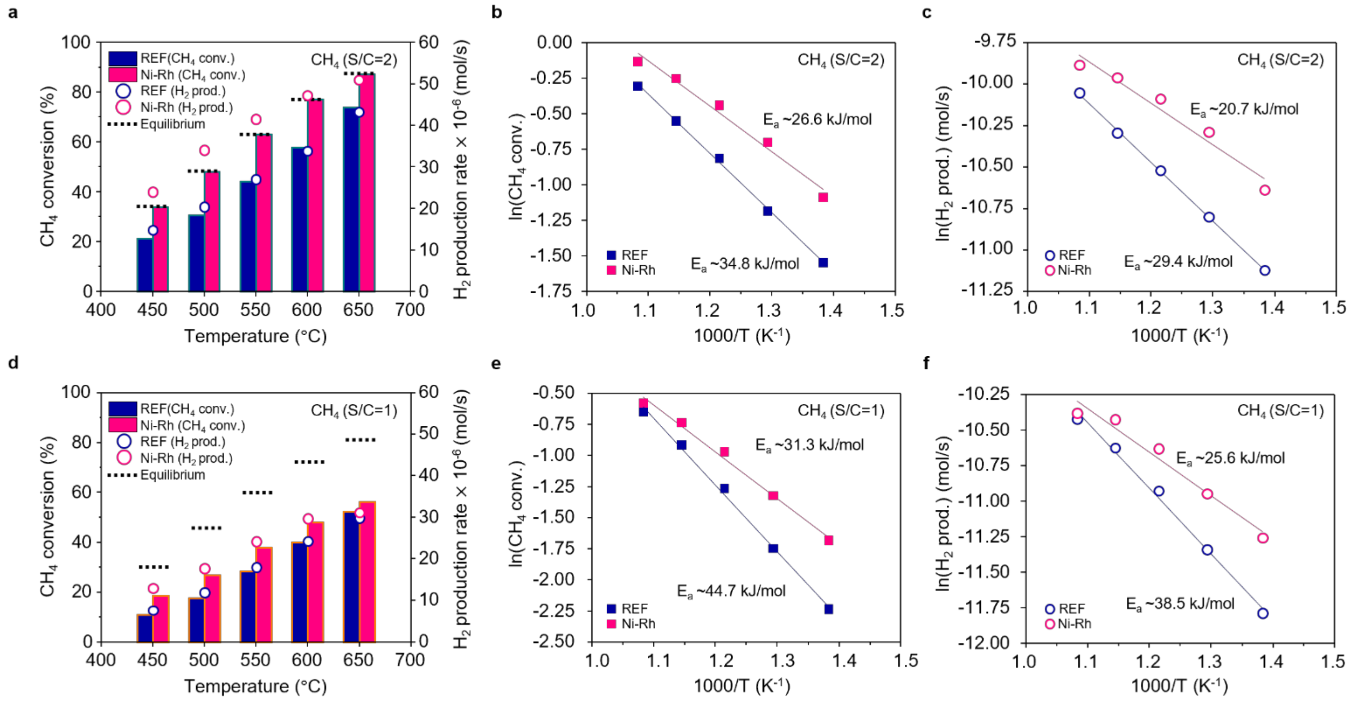

Supplementary Figure 10. Gas molar ratio at equilibrium for steam to carbon ratio of 2 (a), and 1 (b).

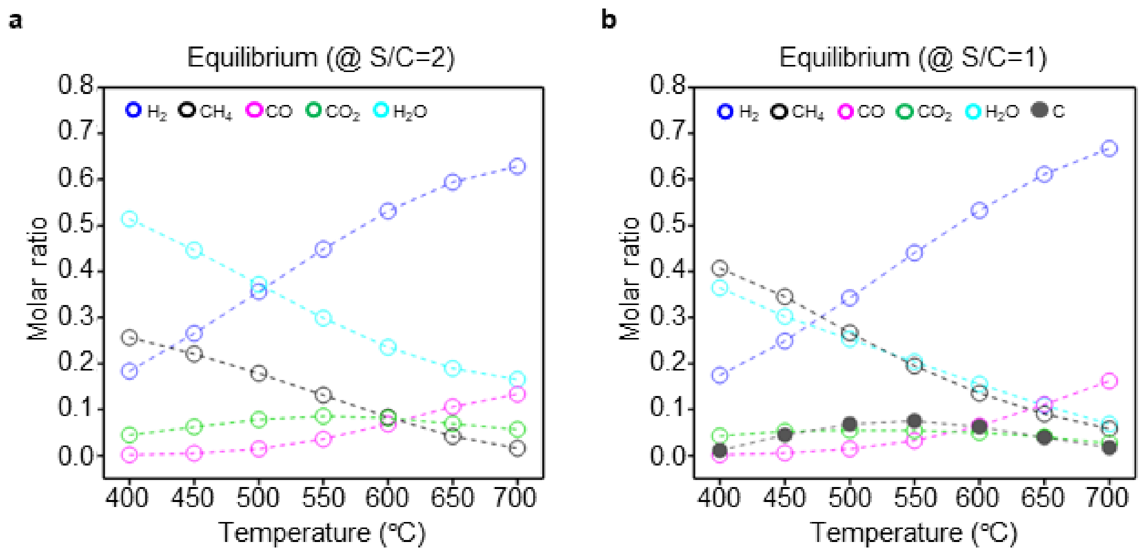

Supplementary Figure 11. Gas composition during cell operation under the CH_4-fueled operation (S/C=2, S/C=1) in the temperature range of 650-450 °C at OCV by gas chromatography. (a) raw data based on dry gas; and (b) calculated data including H_2O gas by C-H-O balance. The fuel conditions for fuel electrode are 25% CH_4, 50% H_2O and 25% Ar for S/C=2, and 25% CH_4, 25% H_2O and 50% Ar for S/C=1, respectively, under same flow rate of 32 sccm.

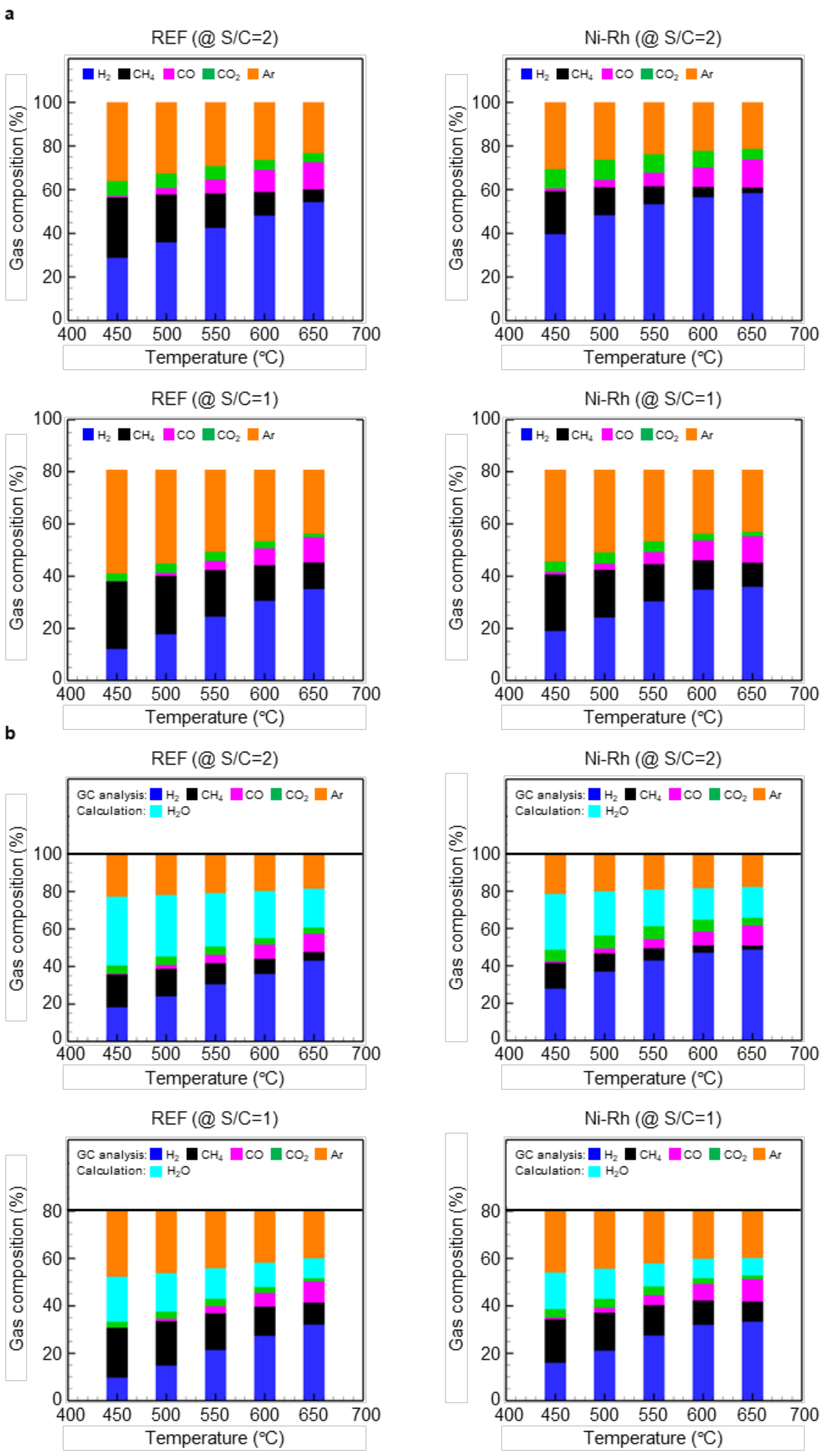

Supplementary Figure 12. Comparison of maximum power densities for P_{H2} and CH_4 fuel operation as a function of partial pressure of H_2 and temperatures (650–450 °C). (a) REF under H_2, (b) Ni-Rh under H_2, (c) REF under CH_4/H_2O (S/C=2), (d) Ni-Rh under CH_4/H_2O (S/C=2), (e) REF under CH_4/H_2O (S/C=1), and (f) Ni-Rh under CH_4/H_2O (S/C=1). The fuel conditions for fuel electrode are 25% CH4, 50% H2O and 25% Ar for S/C=2, and 25% CH4, 25% H2O and 50% Ar for S/C=1, respectively, under same flow rate of 32 sccm.

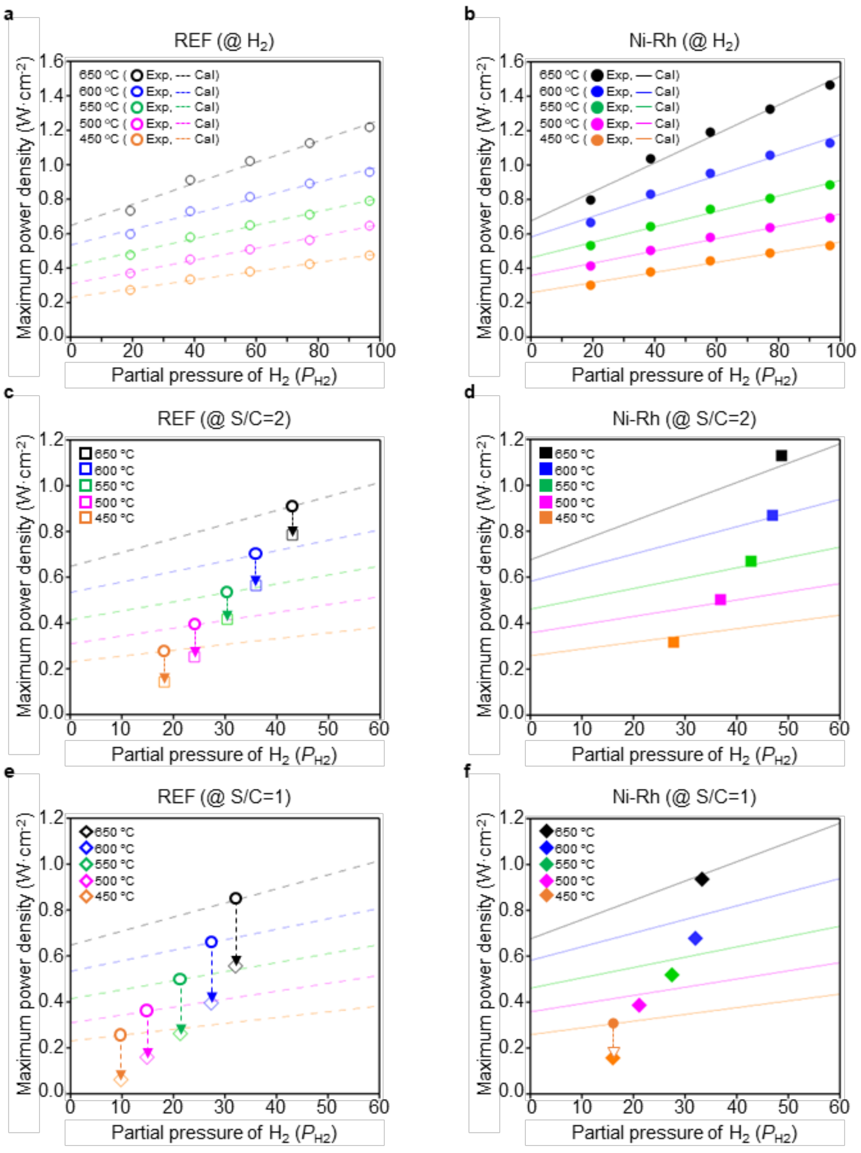

Supplementary Figure 13. EIS analysis of (a) REF and (b) Ni-Rh cell over long-term performance evaluations operated with a fuel composition of 25% CH_4, 25% H_2O and 50% Ar at the fuel electrode with a total flow rate of 100 sccm and air at the cathode as an oxidant (100 sccm).

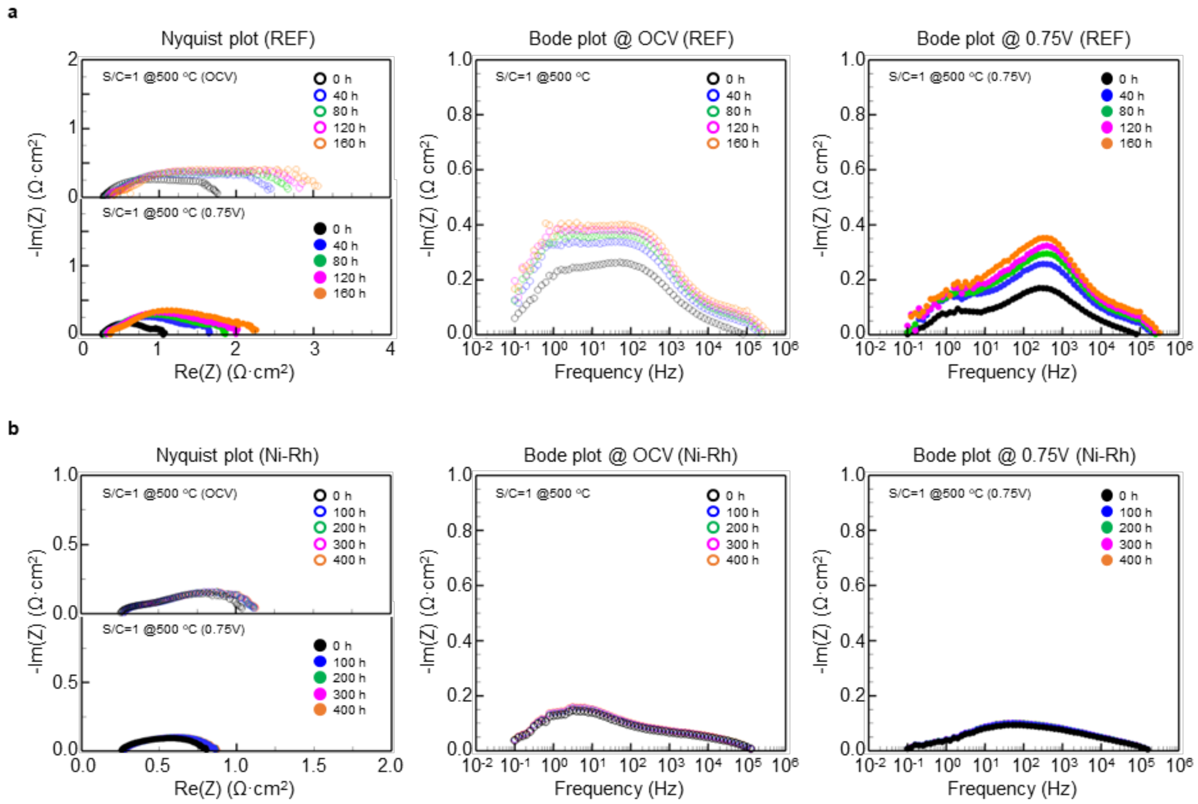

Supplementary Figure 14. Postmortem analysis with EDS spectrum of REF cell (a) and Ni-Rh cell (b) and magnified images of Ni-Rh cell surface (c) by SEM after long-term stability test at 500 °C for 500 h.

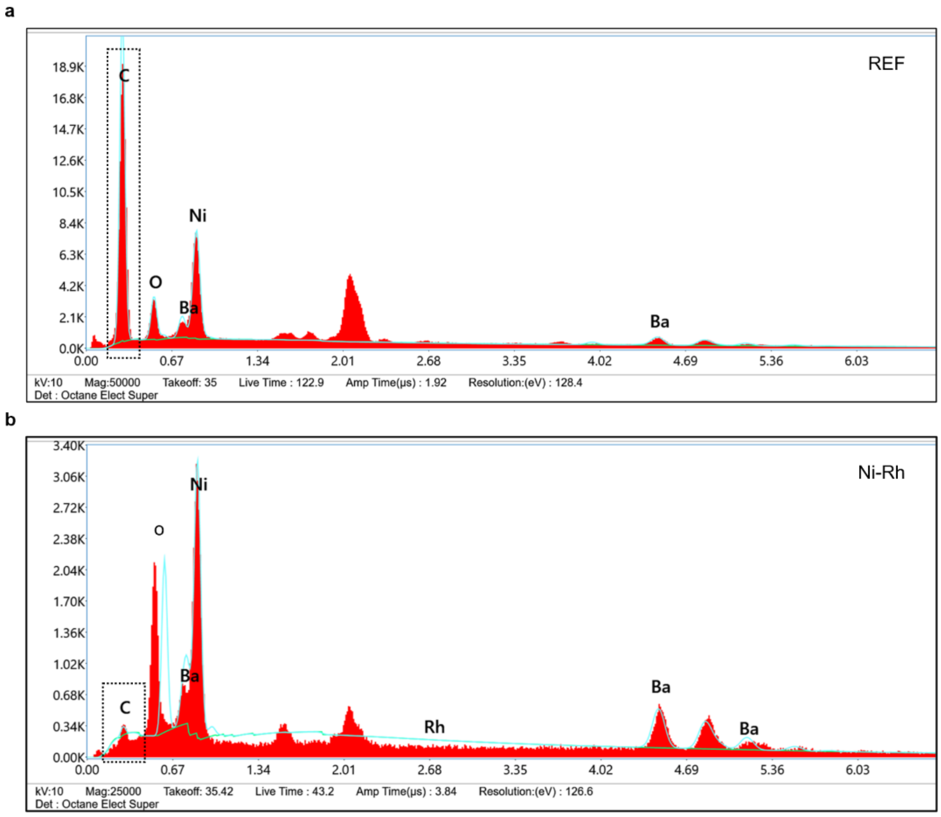

Supplementary Figure 15. Postmortem analysis by Raman for REF and Ni-Rh cells after long-term stability test at 500 °C for 500 h.

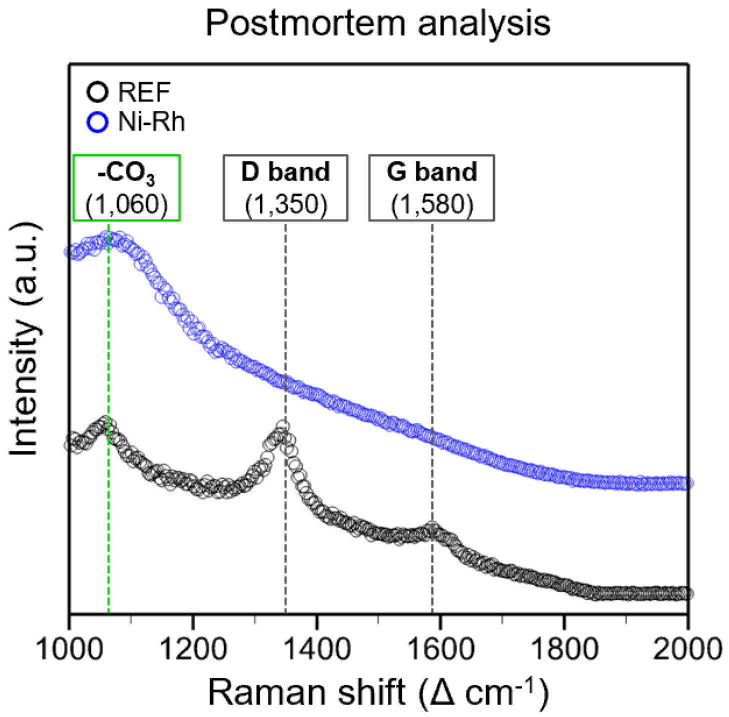

Supplementary Figure 16. Confirmation of thermal stability and carbon-coking tolerance for the Ni-Rh bimetallic catalyst after long-term stability test at 500 °C for 500 h by postmortem analysis with SEM.

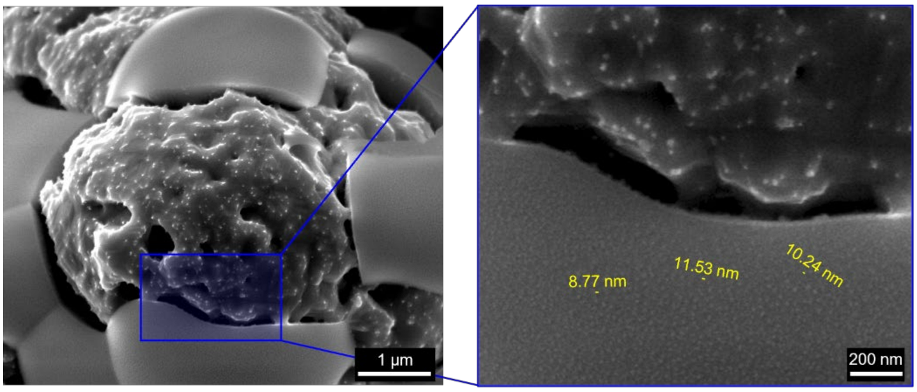

Supplementary Note 3.

Self-carbon cleaning mechanism by in-situ DRIFTS data measured on the fuel electrode surface

In-situ DRIFTS experiment was performed to define the species evolved on the fuel electrode surface of PCFCs during the steam reforming of methane (SRM) reaction, as shown in Supplementary Fig. 17. C=C stretching vibration (C-C bonding, 1515 cm^{-1}) and C-H deformation vibration (CH*, 1340 cm^{-1}) related to carbon formation are common to REF and Ni-Rh, and their peak intensities in Ni-Rh are smaller than those in REF[13]. The results of carbon formation are consistent with the catalytic activity trends for long-term stability results. On the other hand, except for peaks related to carbon formation, other peaks such as formyl, methyl and hydroxyl are higher in Ni-Rh than REF. It can be seen that Ni-Rh is easier to form CO* or CO_2* than REF through the formation of C=O stretching mode (bridged-bonded CO_B*, 1691 cm^{-1})[14] and C-H deformation vibration (CHO*, 1420–1370 cm^{-1})[13]. Ni-Rh is superior to REF for methyl (CH_X*) dissociation through another C-H deformation vibration (CH_X*, 1365–1295 cm^{-1})[13] peak, which is equivalent to the catalytic activity trend related to methane reforming. Moreover, Ni-Rh has independent peaks such as CHO*, CHOH*, CO_T* that do not present in REF. In Supplementary Fig. 17, the C=O stretching vibration (CHO*, 1437 cm^{-1})[14] peak is related to the C-H deformation vibration for CHOH* (1440–1400 cm^{-1})[13], indicating that Ni-Rh forms a CHOH* species by self-carbon cleaning unlike REF and leads to CHO*. The C=O stretching mode (tilted CO_T*, 1664 cm^{-1}) is formed on the surface containing Rh, which can form the other carbonyl species[15]. Furthermore, some peaks of Ni-Rh are greater than that of REF for CH_3-metal groups due to CH_2 rocking vibration (CH_3–M*, 900–700 cm^{-1}), C–O stretching vibration (CO*, 870–850 cm^{-1}), and O–CO in-plane deformation vibration (COOH*, 675–590 cm^{-1})[13]. This indicates that Ni-Rh forms more COOH*, an intermediate species for CO or CO_2 production, than carbon formation from C* contained in CH_4. Therefore, the Ni-Rh bimetallic catalyst has higher carbon resistance than REF through the self-carbon cleaning process, as shown in Supplementary Fig. 18.

Supplementary Figure 17. In-situ DRIFTS studies of the fuel electrode for self-carbon cleaning mechanism at (a) REF and (b) Ni-Rh, respectively, in the temperature range of 100–500 °C. The fuel conditions for the fuel electrode are 3% CH_4, 3% H_2O and 94% Ar for S/C=1 with the flow rate of 20 sccm.

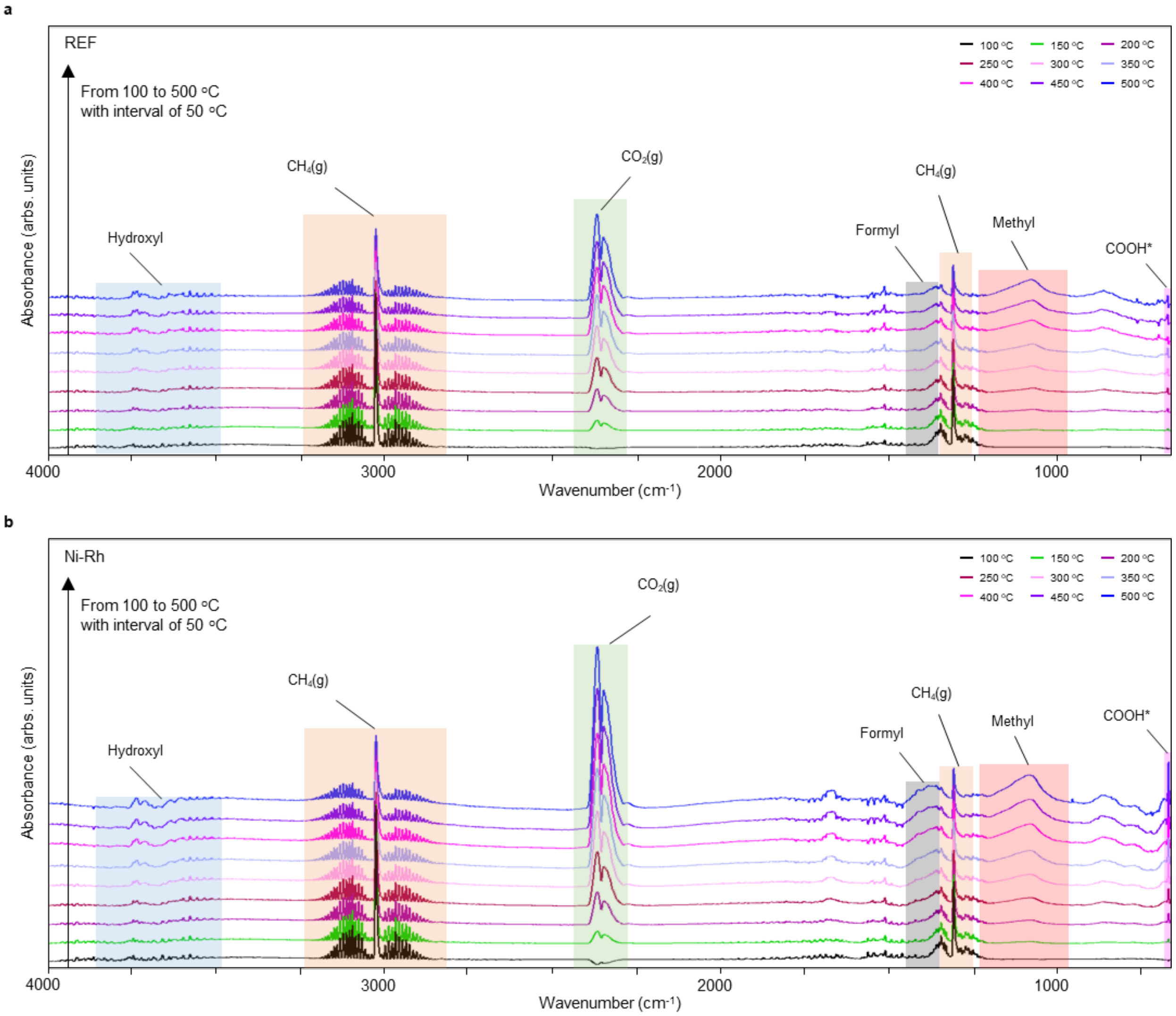

Supplementary Figure 18. Expected reaction pathway of steam reforming of methane for (a) REF and (b) Ni-Rh through in-situ DRIFTS analysis.

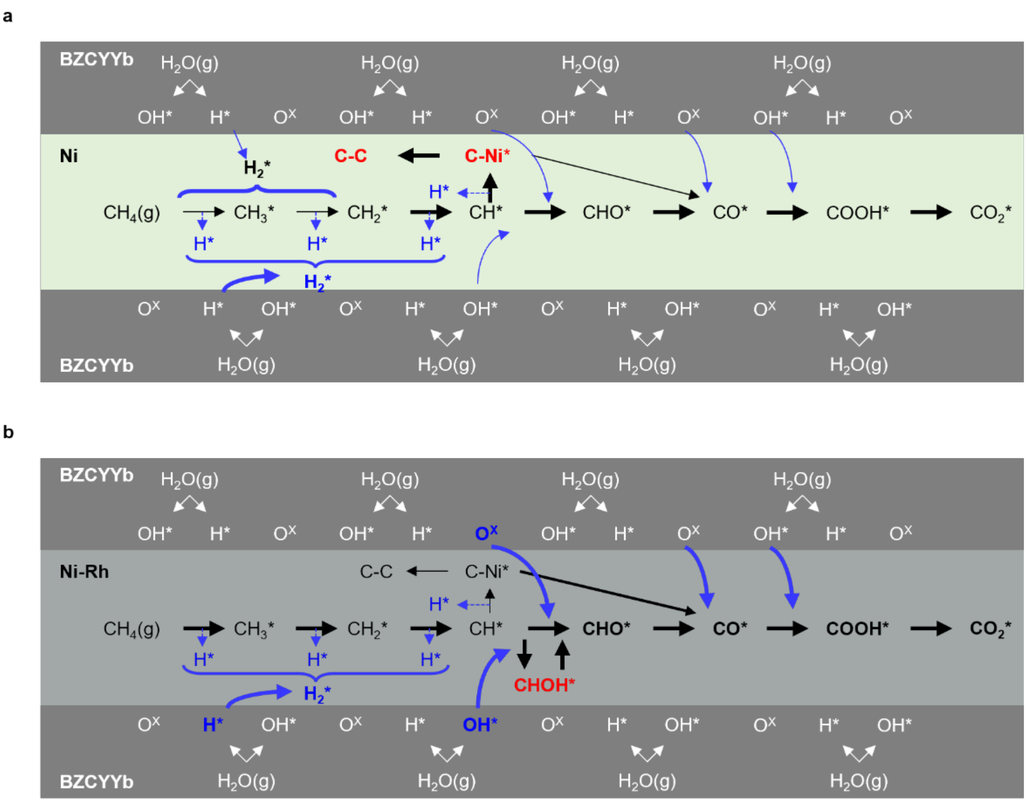

Supplementary Note 4.

Define the specific peak by in-situ XPS data measured on the fuel electrode surface

The specific peaks defined are as follows. C *1s*: C-C sp^3 (285.3 eV, carbon), C-Ni (283.9 eV, carbon precursor), Ads. CO (298.5 eV, adsorbed CO), and Ce *4s* (290.5 eV)[16,17]. Especially, C-Ni (carbon precursor), carbon formed on the Ni surface, changes from Ni-rich film state (283.9 eV) to C-rich film state (284.5 eV) as the carbon content increases, and finally is formed C-C sp^3 (hybridized carbon, 285.3 eV)[16]. O 1s: Vo (530.5 eV, oxygen vacancy), OH* (531.8 eV, hydroxyl group), $O_O^{\times}$ (529.2 eV, lattice oxygen), and Ads. H_2O (532.9 eV, adsorbed H_2O)[17,18]. Ni *2p*: Ni (852.7 eV, Ni metallic state), NiO (853.9 eV, Ni oxide), and $Ni(OH)_2$ (856.3 eV, Ni hydroxyl)[19,20]. All XPS results of samples are normalized by Ce *4s* to compare the area ratio for REF and Ni-Rh cells.

Dry samples (REF and Ni-Rh cells) were prepared without wet pre-treatment and measured at clean surface (after Ar sputtering at room temperature) and reacted surface (after injected CH_4 gas at 500 °C) by in-situ XPS experiment as shown in Supplementary Fig. 19 to compare the effects of H_2O (wet samples). In a dry environment, there is little difference between the results of the REF cell and the Ni-Rh cell in C *1s*, O *1s*, and Ni *2P* photoelectron spectra. In particular, rapid carbon formation (C-C sp^3) is seen after the reaction in C *1s* photoelectron spectra. On the other hand, in Supplementary Table 4, the C *1s* photoelectron spectra result of the REF cell in the wet environment shows carbon formation about 2.5 times after the reaction, but that of the Ni-Rh cell is insignificant in the C *1s* photoelectron spectra. This is because the Ni-Rh cell in the wet environment removed carbon by supplying more Vo, OH* and NiO than the REF cell compared to the results in the dry environment.

Supplementary Figure 19. In-situ XPS studies of the fuel electrode for dry environment feeding CH_4 fuel. The C *1s* photoelectron spectra by comparison of the initial state (room temperature) and CH_4 feeding condition (500 °C) (a) with relative area ratio (b). The O *1s* photoelectron spectra at the initial state (c) with concentration of oxygen vacancy (Vo) and hydroxyl group (OH*) (d). The Ni *2p* photoelectron spectra at the initial state (e) with concentration of NiO (f).

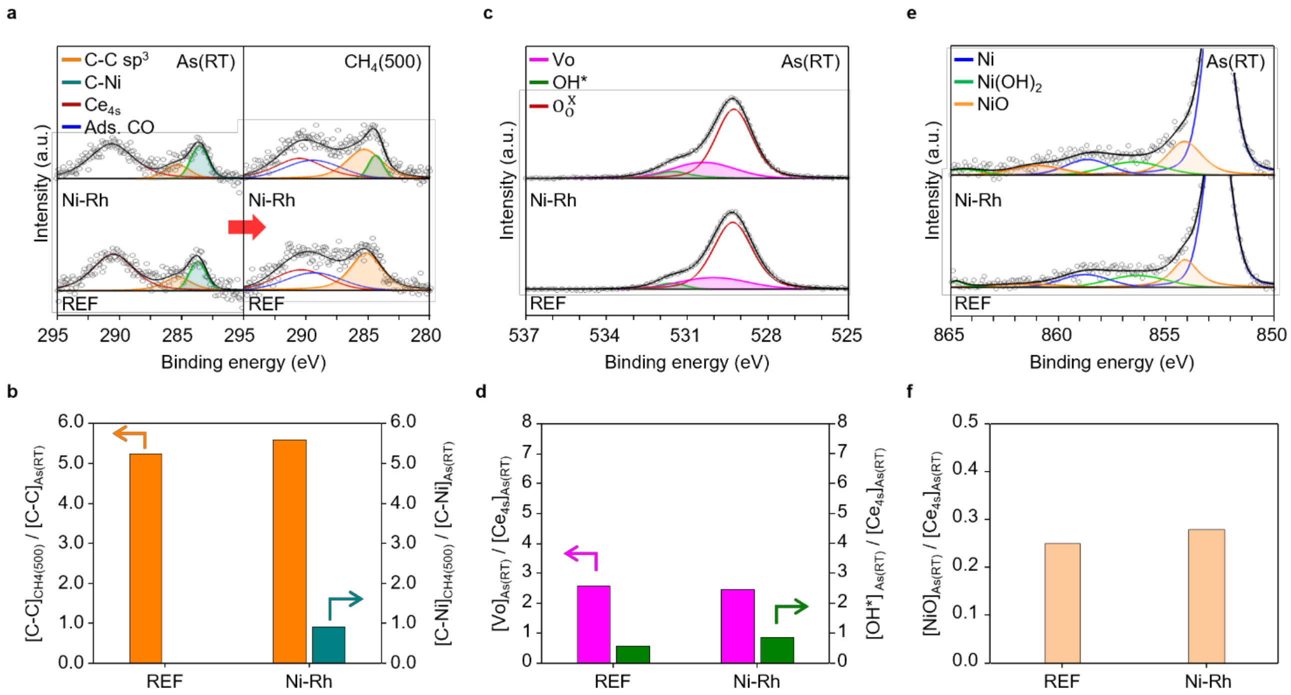

Supplementary Table 4. The comparison of wet and dry environment for relative area ratio value by in-situ XPS analysis.

**Environment**	**$[C\text{-}C]_{CH4(500)}$ / $[C\text{-}C]_{As(RT)}$**<br>REF / **Ni-Rh**	**$[V_o]_{As(RT)}$ / $[Ce_{4s}]_{As(RT)}$**<br>REF / **Ni-Rh**	**$[OH^*]_{As(RT)}$ / $[Ce_{4s}]_{As(RT)}$**<br>REF / **Ni-Rh**	**$[NiO]_{As(RT)}$ / $[Ce_{4s}]_{As(RT)}$**<br>REF / **Ni-Rh**
**Wet**	2.58 / **1.15**	3.59 / **6.60**	2.00 / **3.52**	0.28 / **0.42**
Dry	5.10 / 5.74	2.74 / 2.48	0.57 / 0.84	0.25 / 0.28

Supplementary References

1 Thieu, C.-A. *et al.* Effect of secondary metal catalysts on butane internal steam reforming operation of thin-film solid oxide fuel cells at 500–600° C. *Applied Catalysis B: Environmental* **263**, 118349 (2020).

2 Duan, C. *et al.* Highly durable, coking and sulfur tolerant, fuel-flexible protonic ceramic fuel cells. *Nature* **557**, 217-222 (2018).

3 Duan, C. *et al.* Readily processed protonic ceramic fuel cells with high performance at low temperatures. *Science* **349**, 1321-1326 (2015).

4 Bian, W. *et al.* Revitalizing interface in protonic ceramic cells by acid etch. *Nature* **604**, 479-485 (2022).

5 Chen, Y. *et al.* A robust fuel cell operated on nearly dry methane at 500° C enabled by synergistic thermal catalysis and electrocatalysis. *Nature Energy* **3**, 1042-1050 (2018).

6 Hwang, S. H., Kim, S. K., Nam, J.-T. & Park, J.-S. Triple-component composite cathode for performance optimization of protonic ceramic fuel cells. *International Journal of Hydrogen Energy* **46**, 33551-33560 (2021).

7 Konwar, D. & Yoon, H. H. A methane-fueled SOFC based on a thin BaZr 0.1 Ce 0.7 Y 0.1 Yb 0.1 O 3− δ electrolyte film and a LaNi 0.6 Co 0.4 O 3 anode functional layer. *Journal of Materials Chemistry A* **4**, 5102-5106 (2016).

8 Lei, L., Keels, J. M., Tao, Z., Zhang, J. & Chen, F. Thermodynamic and experimental assessment of proton conducting solid oxide fuel cells with internal methane steam reforming. *Applied Energy* **224**, 280-288 (2018).

9 Suzuki, T. *et al.* A functional layer for direct use of hydrocarbon fuel in low temperature solid-oxide fuel cells. *Energy & Environmental Science* **4**, 940-943 (2011).

10 Zha, S., Moore, A., Abernathy, H. & Liu, M. GDC-based low-temperature SOFCs powered by hydrocarbon fuels. *Journal of The Electrochemical Society* **151**, A1128 (2004).

11 Hong, K., Sutanto, S. N., Lee, J. A. & Hong, J. Ni-based bimetallic nano-catalysts anchored on BaZr 0.4 Ce 0.4 Y 0.1 Yb 0.1 O 3− δ for internal steam reforming of methane in a low-temperature proton-conducting ceramic fuel cell. *Journal of Materials Chemistry A* **9**, 6139-6151 (2021).

12 Zhu, T. *et al.* Microkinetics of steam methane reforming on platinum and rhodium metal surfaces. *Journal of catalysis* **297**, 227-235 (2013).

13 Socrates, G. *Infrared and Raman characteristic group frequencies: tables and charts*. (John Wiley & Sons, 2004).

14 Sun, W. *et al.* V-Doped Cu2Se Hierarchical Nanotubes Enabling Flow-Cell CO2 Electroreduction to Ethanol with High Efficiency and Selectivity. *Advanced Materials*, 2207691 (2022).

15 Chen, L. *et al.* Carbon monoxide-free hydrogen production via low-temperature steam reforming of ethanol over iron-promoted Rh catalyst. *Journal of Catalysis* **276**, 197-200 (2010).

16 Furlan, A., Lu, J., Hultman, L., Jansson, U. & Magnuson, M. Crystallization characteristics and chemical bonding properties of nickel carbide thin film nanocomposites. *Journal of Physics: Condensed Matter* **26**, 415501 (2014).

17 Salcedo, A. *et al.* Reaction Pathway for Coke-Free Methane Steam Reforming on a Ni/CeO2 Catalyst: Active Sites and the Role of Metal–Support Interactions. *ACS catalysis* **11**, 8327-8337 (2021).

18 Klauke, K. *et al.* Enhancement of the SrTiO3 surface reactivity by exposure to electric fields. *ChemNanoMat* **5**, 948-956 (2019).

19 Peck, M. A. & Langell, M. A. Comparison of nanoscaled and bulk NiO structural and environmental characteristics by XRD, XAFS, and XPS. *Chemistry of Materials* **24**, 4483-4490 (2012).

20 Chastain, J. & King Jr, R. C. Handbook of X-ray photoelectron spectroscopy. *Perkin-Elmer Corporation* **40**, 221 (1992).
